# Core build-up resin composites: an in-vitro comparative study

**DOI:** 10.1080/26415275.2020.1838283

**Published:** 2020-11-03

**Authors:** L. Spinhayer, A.T.B. Bui, J.G. Leprince, C.M.F. Hardy

**Affiliations:** a School of Dental Medicine, Cliniques Universitaires Saint-Luc, UCLouvain, Brussels, Belgium; b DRIM research group, Advanced Drug Delivery and Biomaterials, Louvain Drug Research Institute (LDRI), UCLouvain, Brussels, Belgium

**Keywords:** core build-up, resin composite, dual cure, flexural modulus, flexural strength

## Abstract

**Aim:**

Resin composite (RC) are commonly used under full crowns. However, independent information is lacking to guide practitioners regarding core RC material selection. This study aimed at comparing the flexural properties of a large selection of commercially-available core build-up RCs (CBU-RC), either light-, self- or dual-cure, to conventional light-cure RCs.

**Methods:**

RCs were injected into a 25 × 2×2mm Teflon mold, and either light-cured during 20 s (materials with claimed light-cure characteristics) or covered by aluminum during 10 min (dual- and self-cure CBU-RCs). They were subjected after a one-week water storage at 37.5 °C to three-point bending, and Flexural modulus (*E*_flex_) and Flexural Strength (*σ*_f_) were calculated (*n* = 20). Thermogravimetric analysis (*n* = 3) was performed to determine inorganic filler content (%).

**Results:**

For dual-cure CBU-RCs, both RC (*p* < .0001) and light-curing (*p* = .0007) had a significant influence on *E*_flex_, while only RC was significant for *σ*_f_ (*p* < .0001). Between all conventional RCs and CBU-RCs, significant differences were observed (*p* < .0001), both regarding *E*_flex_ and *σ*_f_, with values ranging from 3.9 to 15.5 GPa and from 76 to 130.3 MPa, respectively. Higher *E*_flex_ values were observed for light-cure RCs than for self- and dual-cure ones, while no clear trend was noticed regarding *σ*_f_. Good linear correlation was found between inorganic filler content and *E*_flex_ (*R*^2^=0.85, *p* < .0001), but not with *σ*_f_ (*R*^2^=0.08, *p* = .1609).

**Conclusion:**

This work demonstrated a positive influence of light-curing on dual-cure CBU-RC’s *E*_flex_. It also highlighted large differences in flexural properties (especially *E*_flex_) among the investigated materials, questioning the use of some CBU-RCs as dentin replacement in case of large tissue loss.

## Introduction

Strategies for restoring severely damaged teeth have changed considerably over the last few years with the advent of adhesive dentistry [1]. Dentistry is evolving to become less and less invasive, the preservation of dental tissues becoming a major concern for practitioners [2]. Indirect bonded restorations are therefore more and more common and reliable [3,4] Nevertheless, full crowns remain a valid option, with long survival rates [4], and are particularly indicated for specific indications, i.e. bridges, crown with precision attachment for removable prosthesis, crown replacement, or highly discolored teeth in need of coverage. In any case, the teeth requiring a full crown are often severely damaged, with large structural loss and, as a result, often root canal-treated. Whether the root canal treatment additionally weakens the tooth is debated [5,6], but most agree that the loss of tooth structure at the coronal level represents the major cause for increased risk of tooth fracture [7]. The low amount of remaining tooth tissue represents a major restorative challenge for the practitioner to ensure the best possible longevity of both the restoration and the tooth. The classical restorative strategy, supported by many clinical studies, consists of preparing a core build-up, with or without a root canal post, followed by the placement of a full crown.

Various strategies are available regarding the core build-up, the oldest of which being a direct core build-up using amalgam. The latter presents good mechanical properties and clinical performances and is still described by some authors as one of the best options for core build-ups with extensive tissue loss [8,9]. However, this material is progressively disappearing from dental practices for various reasons, including aesthetic, political and environmental considerations, as well as the lack of adhesion to tooth tissues. Another possibility is the custom metallic cast post and core, which is clinically well established and remains another valid option in terms of restoration longevity, but which has been associated with more dramatic failure modes [10]. They also require longer clinical and lab time and involve additional costs. For these reasons along with the progress made over the years in adhesive technology [11], resin composites (RCs) are more and more used by practitioners as core build-up materials [5]. These restorations save time for the practitioner, who can build the core in one go and one appointment and is less expensive for the patient.

However, there is a lack of recent independent information to guide practitioners regarding RC core material’s selection [12]. Moreover, among the large selection of RC core materials commercially available, three different curing modes exist, i.e. self-, light- and dual-curing. The latter has been introduced to combine the fast and on-demand setting of light-curing materials, as well as self-cure characteristics in order to compensate for the lack of accessibility of photons in deep cavities, especially in the pulp chamber and in the root canal when placing a post. However, the benefit of light exposure in dual-cure RC to reach maximal polymerization has been highlighted for luting resin composites [13–18]. Hence, the impact of self- and light-curing modes needs to be critically evaluated for core RC materials.

The latter representing a substantial part of the substrate supporting the crown, their flexural properties should be as close as possible to those of the dentin to reduce interfacial stress generation [19,20] and the risk of fracture in endodontically-treated teeth [7,21].This study therefore aimed at comparing the flexural properties of a large selection of commercially-available core build-up RC (CBU-RC), either light-, self- or dual-cured, to conventional light-cure RC and to the values reported for dentin.

The objectives of the work were translated into two null hypotheses with regards to the materials’ flexural properties: (1) There is no significant difference between the dual-cure CBU-RC with or without light-curing; (2) there is no significant difference between investigated RCs.

## Materials and methods

Twelve CBU-RC were tested and compared to five conventional light-cure RCs (Table 1). The resin composites were injected into a 25 × 2×2mm white Teflon split-mold and covered on both sides by a Mylar strip to minimize oxygen inhibition. They were then either light-cured during 20 s (all light-cure materials, *n* = 20) or covered during 10 min by an aluminum foil directly after the mixing process (dual-cure and self-cure CBU-RC, *n* = 20). These light- and self-curing durations (20 s and 10 min, respectively) were arbitrarily chosen both to standardize for all materials and to avoid any undercuring due to a lack of respect of manufacturers recommendations; these durations were indeed similar to or higher than the recommended durations of all materials (Table 1). The light-curing was performed using the Bluephase G2 polywave light (Ivoclar Vivadent, Schaan, Liechtenstein) set in ‘high-power’ curing mode (irradiance = 1100mW/cm^2^) in 3 non-overlapping spots, in contact with the Mylar strip, starting in the middle of the sample. After curing, each sample was polished with P#500 and then P#1000 discs (Struers^®^ Silicon Carbide Waterproof Paper FEPA). They were then placed for one week in a distilled water bath at 37.5 °C in a temperature-controlled oven (Memert^®^ model 100–800) prior to testing. Before the mechanical tests, the samples were removed from the bath and dried. Their dimensions were measured by a graduated caliper (Mitutuyo^®^ Absolute Digimatic) and validated (2 ± 0.5 mm × 2 ± 0.5 mm × 25 ± 0.5 mm). When the presence of major defects in the form of air bubbles was observed before being tested, the sample was discarded and replaced to keep the same total number of samples per condition.

**Table 1.** Characteristics of the resin-based composites tested according to manufacturer data when available.

	Manufacturer	Product name	Type	Inorganic fillers / particle size	Curing type	Setting time	Working time	Flexural strength	Indications
Conventional resin composites	3M ESPE	Filtek Supreme XTE	Micro-hybrid / Nano-filled	50 vol% (71 wt%) / 0.02 µm to 2.4 µm	Light-cure	NA	NA	NP	Core Build-up, anterior, posterior, direct, indirect restoration, contention.
	Ivoclar-Vivadent	Tetric Evoceram	Micro-hybrid / Nano-filled	75–76 wt.% or 53–55 vol.%	Light-cure	NA	NA	120 MPa	Restoration of deciduous teeth – anterior, posterior et cervical regions, veneering of discoloured anterior teeth, splinting of mobile teeth, extended fissure sealing in molars and premolars, repair of composite/ceramic veneers, build-ups for transparent, removable Invisalign® orthodontic retainers.
	Kuraray	Clearfil APX	NP	71 wt.% / 0.02 µm to 17 µm	Light-cure	NA	NA	NP	Posterior anterior tooth, root surface defect.
	Kuraray	Clearfil Majesty Post	Micro-hybrid / Nano-filled	82wt.% / 0.02 µm to 7.9 µm	Light-cure	NA	NP	177 MPa	Posterior restorations.
	VOCO	Grandio	Micro-hybrid / Nano-filled	71.4 wt.% (87 wt.%)	Light-cure	NA	NP	161 MPa	Class I to V fillings, traumatically affected anteriors, facetting of discoloured anteriors.
Core build-up resin composites	Dentsply	Core X-Flow	Micro-hybrid / Nano-filled	NP	Dual-cure	2–3'	40′'–1′30′'	120 MPa	Core Build-up, indirect restoration, adhesive cementation of fiber posts.
	DMG	Luxacore Z Dual	Hybrid	NP	Dual-cure	5′	1'30’	150 MPa	All reconstructions.
	GC	Gradia Core	NP	NP	Dual-cure	5′	3′ at 23 °C		Core build-up and fiber posts cementation.
	Ivoclar-Vivadent	Multicore Flow	Hybrid	46 vol% (70 wt%) / 0.04 µm to 2 µm	Dual-cure	4-5 '	90-120''	95 MPa	Core Build-up and adhesive cementation of glass fiber posts.
	Kuraray	Clearfil DC Core Plus	Hybrid	52wt.% / 0.01 µm to 20 µm	Dual-cure	6′	3′	107 MPa	Core build-up, adhesive cementation of fiber posts.
	Kuraray	Clearfil Photocore	Hybrid	68 wt.% / 0.49 µm to 75 µm	Light-cure	NA	5′	125 MPa	Core build-up.
	Septodont	N'Durance Dimer Core	Micro-hybrid / Nano-filled	58 vol% / 0.01 µm to 13 µm	Dual-cure	4′	2′	*E* = 5,5 GPa	Direct core build-up under crowns, bridges, inlay/onlay.
	Ultradent	PermaFlo	NP	68 wt.% / 0,7µm	Dual-cure	5′ à 8′	2'30′'	NP	Anterior and posterior restorations, lower layers when using incremental building of composites.
	VOCO	Grandio Core	Micro-hybrid / Nano-filled	77 wt.%	Dual-cure	5′	1'30’	125 MPa	Core buil-up.
	VOCO	Rebilda DC	Hybrid	71 wt.%	Dual-cure	5′	2′	102 MPa	Adhesive core build-up on vital and non-vital teeth, luting of fibre-reinforced resin posts.
	VOCO	Rebilda LC	Hybrid	77 wt.%	Light-cure	NA	NP	NP	Core build-up on vital and non-vital teeth and blocking out.
	VOCO	Rebilda SC	Hybrid	70 wt.%	Self-cure	NP	NP	57 MPa	Adhesive core build-up on vital and non-vital teeth, luting of fibre-reinforced resin posts

NA: not applicable; NP: not provided.

The flexural modulus (*E*_flex_) and flexural strength (*σ*_f_) were calculated after using a three-point bending test. Samples (*n* = 20) were placed in a universal testing machine (Instron 5566, High Wycombe, UK) with a distance of 20 mm between the supports and loaded at a cross- head speed of 0.75 mm/min until fracture occurred, based on ISO4049 recommendations.

Thermogravimetric analysis (TGA) (*n* = 3) (Mettler Toledo Greifensee, Switzerland) was performed to determinate the inorganic filler content, as described by Randolph et al. [22].

The statistical analyses were performed using JMP software (JMP Genomics; SAS Institute). The normality was verified for *σ*_f_ using a Shapiro–Wilk test. For the inorganic filler content and E_flex_ values, Q–Q plots were used to verify the normality of the residuals (after logarithmic transformation for *E*_flex_).

A two-way ANOVA was performed to study the impact of RC’s type and light-cure specifically for dual-cure materials. A one-way ANOVA followed by *post hoc* Tukey multiple comparison tests (*p* = .05) was performed to compare all RCs with regards to inorganic filler content, *E*_flex_ and *σ*_f_. Linear correlation between the filler content and the *E*_flex_ and *σ*_f_ values were also performed, combined with ANOVA (*p* = .05).

## Results

**Figure 1.**
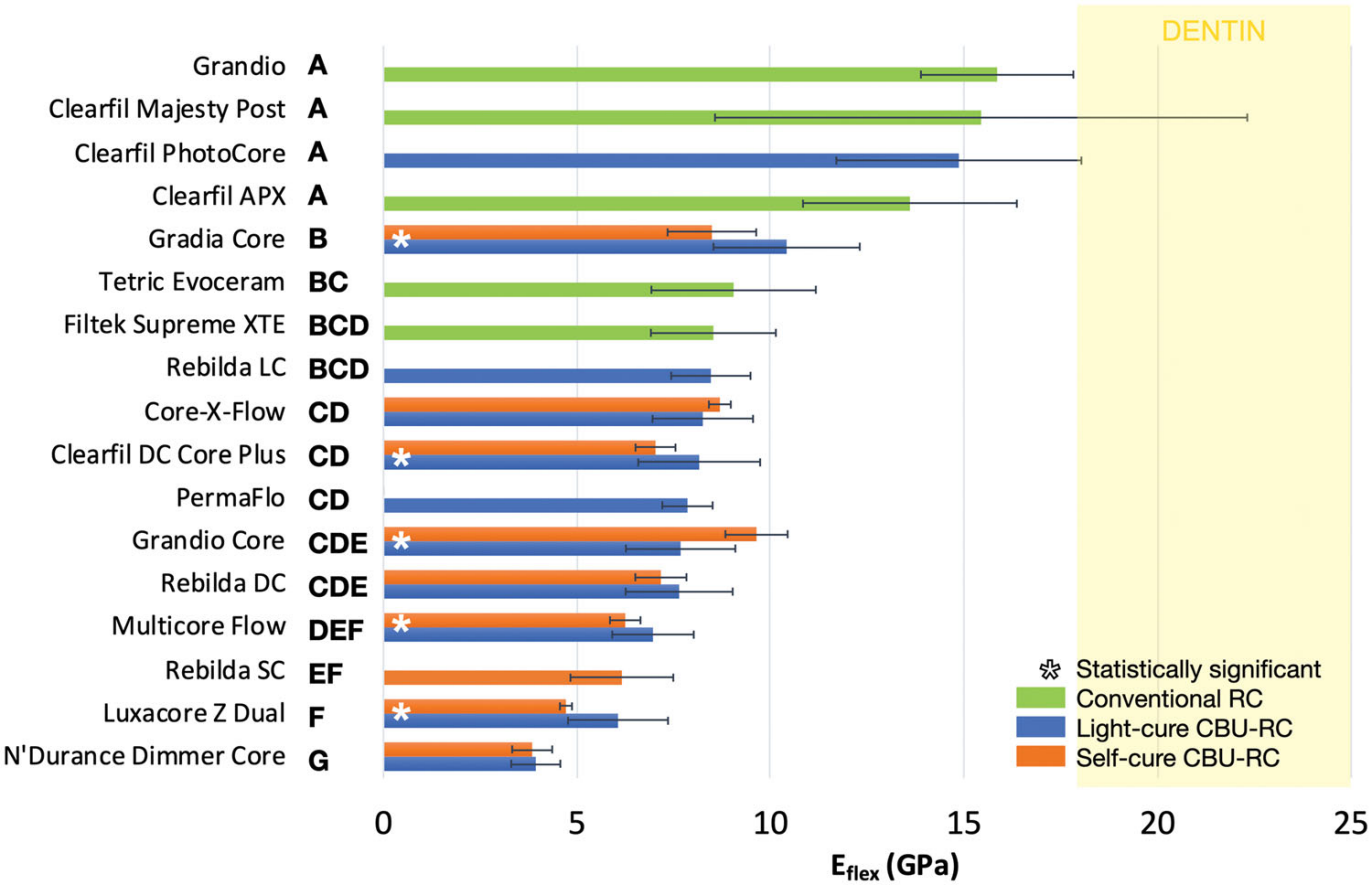
Flexural modulus (E_flex_, GPa) calculated after three-point bending test (*n* = 20). The materials are ranked in descending order based on their average values; standard deviations are added as horizontal whiskers for each histogram. Similar capital letters placed on the right side of the material names connect RCs presenting no statistically significant difference (*p* > .05). Dentin values appear for the sake of comparison: 18–25 GPa [32].

**Figure 2.**
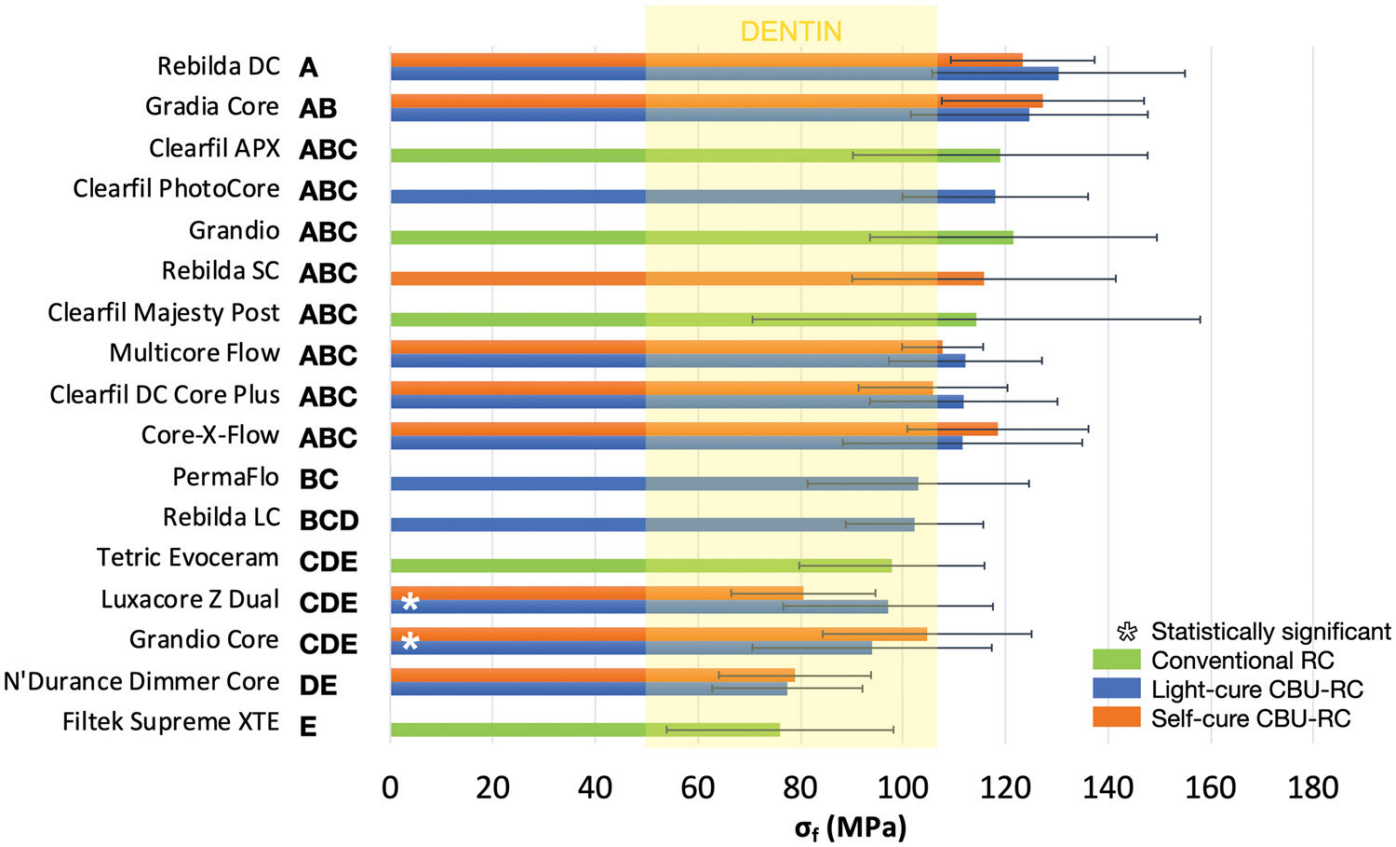
– Flexural strength (*σ*_f_, MPa) calculated after three-point bending test (*n* = 20). The materials are ranked in descending order based on their average values; standard deviations are added as horizontal whiskers for each histogram. Similar capital letters placed on the right side of the material names connect RCs presenting no statistically significant difference (*p* > .05). Dentin values appear for the sake of comparison: 52–105 MPa [32].

For dual-cure CBU-RCs, two-way ANOVA revealed that light-curing had a significant and favorable impact on *E*_flex_ (*p* = 0.0007) but not on *σ*_f_ (*p* = .47), the type of RC affecting significantly both properties (*p* < .0001) (Figures 1 and 2).

**Figure 3.**
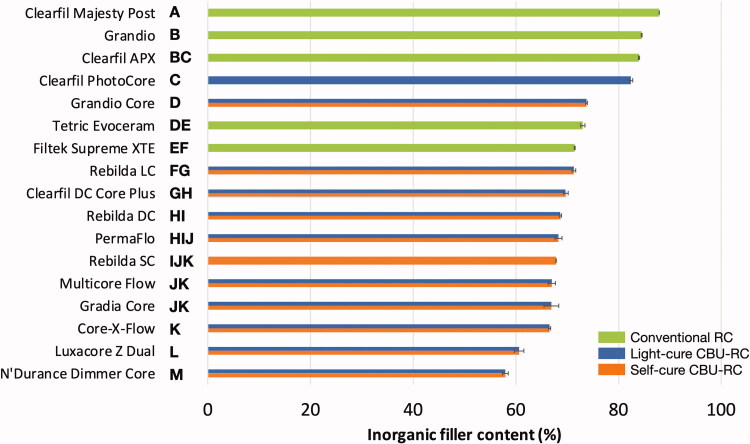
– Inorganic filler content (%) measured by thermogravimetric analysis (*n* = 3). The materials are ranked in descending order based on their average values; standard deviations are added as horizontal whiskers for each histogram. Similar capital letters placed on the right side of the material names connect RCs presenting no statistically significant difference (*p* > .05).

One-way ANOVA revealed significant differences between all RCs both regarding *E*_flex_ and *σ*_f_ (*p* < .0001), with values ranging from 3.9 to 15.5 GPa and from 76 to 130.3 MPa, respectively (Figures 1 and 2). Higher *E*_flex_ values were observed for light-cure RCs than for self- and dual-cure ones, while no clear trend was noticed regarding *σ*_f_. Good linear correlation was found between *E*_flex_ and inorganic filler content (*R*^2^=0.85, *p* < .0001), but not between the latter and *σ*_f_ (*R*^2^=0.08, *p* = .1609). As a result, the values of inorganic filler content (%) followed a very similar trend as those observed for *E*_flex_ (Figure 3). It must finally be mentioned that no values could be obtained for the PermaFlo in self-cure mode, since the samples were still soft after 10 min (and even after 24 h).

## Discussion

The first null hypothesis was rejected since significant differences were observed between self- and light-cure modes of some dual-cure CBU-RCs. The trend observed in the present work in favor of the light-curing mode confirmed previous works based on a smaller number of core RC materials and measuring conversion [23,24] and microhardness [23]. The same trend was also previously described for another category of dual-cure RCs, i.e. luting resin composites [13–17,25]. Light curing is therefore likely to contribute to increase the flexural properties of dual-cure CBU-RCs where it is most needed, i.e. on the coronal aspect. Clinician should therefore take that into account when building up their core, and light-cure their restoration following material injection. However, given the relatively minor drop of flexural properties observed in the self-curing mode for the materials considered, most materials are expected to perform well in the unreachable areas, such as into the root canal. Two notable exceptions were however noticed: PermaFlo and Grandio Core. Since PermaFlo was not able to be tested in self-curing mode as it remained soft. After contacting the manufacturer in this regard, the explanation given by the representative was that a minimum amount of photons is mandatory to initiate the polymerization. No additional explanation was provided. This underlines once more that all materials’ indications should be properly and independently verified. Concerning Grandio Core, the significant difference observed in favor of self-cure condition is unexpected. The specific composition of Grandio Core could explain these results, with an optimization of the self-cure process in this specific material. However, due to the proprietary nature of the composition of commercial materials, this is impossible to investigate further.

The second null hypothesis was also rejected since there were significant differences between the investigated RCs in terms of *σ*_f_ and *E*_flex_.

The lack of clear trend between the various tested materials regarding σ_f_ and the absence of linear correlation with inorganic filler content (*R*^2^=0.08, *p* = .1609) is in line with a recent extensive work on direct resin composites [22]. Several explanatory hypotheses have been suggested, such as differences in resin matrix composition, quality of filler silanization, or the presence of stress-absorbing structures such as nano-clusters. Another suggested explanation was the sensitivity of strength measurement with regard to specimen surface preparation [22], since it is well-known that strength is not an inherent material property but depends on the specimen geometry and preparation [26]. This might then play a part when comparing materials with various injection systems. As mentioned above, it was indeed decided during this work to discard samples and repeat them when the presence of major defects (air bubbles) was observed. Despite optimal conditions and the proper use of double-mix syringes with a thin tip, the presence of such defects could not be avoided. Hence, this is even more likely to occur in clinical conditions, and might further affect the considered properties. In terms of clinical relevance *σ*_f_ has been moderately correlated with clinical wear, especially after solvent storage prior to testing [27], which was done in this study. However, such correlation was highlighted in the context of direct restorations, which does not seem relevant regarding the use of RCs in core build-ups, since the materials are ultimately covered with a crown. While strength has been described as one major criteria for core material selection [9], it might in fact not be as critical on the coronal aspect, especially since surface defects could be filled with the luting material.

Contrary to *σ*_f_, *E*_flex_ is an intrinsic material property, and the strong correlation observed between inorganic filler load and *E*_flex_ is in line with several previous works [22,28,29]. The fact that light-cure RCs occupy the upper half ranking of *E*_flex_ values as compared to self- and dual-cure RCs can therefore be explained by their higher filler content. The necessary bi-component formulation of self- and dual-cure materials limits their filler content, as too large an increase in filler content would prevent their proper mixing due to an excessive viscosity.

The exact clinical consequences of using a CBU-RC with lower *E*_flex_ is difficult to predict [30]. However, it has been stated in a recent review on direct RCs that the property for which these materials are the most clearly deficient in comparison to amalgam is specifically *E*_flex_ [30]. This is consistent with the findings of a previous study comparing two commercial RCs and amalgam as core build-up materials [31]. As mentioned above, amalgam has been considered as an excellent option for core build-ups [8,9], and using a material with a much lower *E_f_*_lex_ may lead to increased deformation, especially under high stress [30]. It was also reported in finite element analyses that restoring teeth with materials presenting a much higher [19] or a much lower *E*_flex_ [20] than dentin will lead to increased interfacial stress generation. The importance of *E*_flex_ was further highlighted in a study showing that the use of CBU-RC with a higher *E*_flex_ increased fracture resistance of endodontically-treated teeth restored with fiber post and resin composite build-up [21]. As a result, and despite the greater ease of use of flowable CBU-RCs, it may seem more reasonable to opt for CBU-RCs with *E*_flex_ values as close as possible to those of dentin. This is in agreement with a previous paper on the topic [12]. Based on the present work, filler content seems an easily accessible and reliable information for practitioners to rationalize their CBU-RC selection. *E*_flex_ values of dentin have been evaluated as ranging between 18 and 25 GPa [32], but in the present study only the highly-filled light-cure composites reached even the lowest of these values (Figure 1). However, their lack of self-cure ability precludes their use in areas where light cannot reach them, especially when placing in the root canal. For such use, dual-cure RCs seem most appropriate, even if less rigid [33]. To ‘compensate’ for the lower mechanical properties of CBU-RCs, some practitioners may consider placing a post. Nevertheless, the need to place fiber posts to restore heavily damaged teeth is more and more questioned for several reasons. First, the bonding conditions in the root canal are not favorable because of the high C-factor, the difficulty of drying and the poor arrangement of the dentin fibers [34], resulting in a major reduction of bond strength in the canal beyond the coronal third [35]. Secondly, it has been stated that clinical data are insufficient to justify the need of fiber posts both for the reinforcement of endodontically treated molars [6,36,37] and for the restoration retention [6,38]. This is especially true for teeth with ferrule, which was identified along with the maintenance of cavity walls as the dominant factors as regards both tooth and restoration survival [37,39]. In the presence of a ferrule, the importance of the material used for core build up was described as non-significant [40], as was the presence of a post [41]. In the absence of ferrule, it was stated that poor clinical outcome is to be expected [39]. A recent systematic review presented a trend confirming that statement (88% restoration survival with ferrule vs 78% without ferrule), but without statistically significant differences [42].

Although these aspects are beyond the scope of this work, they help putting the results in perspective. The ease of use of self- and dual-cure CBU-RC is indeed particularly attractive for clinical situations that require post placement and core build-up, since both can in principle be made in one step, with the same material. Nevertheless, since the need for post placement is questioned, the use of dual-cure CBU-RCs is no longer essential, and the choice of highly filled light-cure RCs with the highest *E*_flex_ values appears as the most appropriate choice with regards to mechanical considerations. In the presence of a ferrule, the mechanical properties of the CBU-RCs are expected to be of lower or no importance. On the contrary, the use CBU-RCs with flexural properties lower than dentin may possibly be more deleterious in the absence of ferrule. Finally, if the clinician still wishes to place a fiber post, it is recommended to combine post cementation with a dual-cure RC and core build-up with a highly filled light-cure material.

## Conclusion

This work confirmed the positive influence of light-curing on the *E*_flex_ of dual-cure CBU-RCs. It also highlighted large differences in *σ*_f_ and *E*_flex_ among the investigated materials, especially for the latter, for which only a few highly filled light-cure materials approached dentin values. These materials therefore seem the most appropriate for core build-up. On the contrary, the use of the self- and dual-cure CBU-RCs with lower inorganic filler content for core build-ups is questionable as dentin replacement in case of large tissue loss. Finally, inorganic filler content seems an easily accessible and reliable information for practitioners to select their CBU-RC.
